# Prevalence and correlates of SARS-CoV-2 seropositivity among people who inject drugs in Baltimore, Maryland

**DOI:** 10.1016/j.dadr.2023.100184

**Published:** 2023-08-09

**Authors:** Eshan U. Patel, Shruti H. Mehta, Becky L. Genberg, Owen R. Baker, Catherine G. Schluth, Jacquie Astemborski, Reinaldo E. Fernandez, Thomas C. Quinn, Gregory D. Kirk, Oliver Laeyendecker

**Affiliations:** a Department of Epidemiology, Bloomberg School of Public Health, Johns Hopkins University, Baltimore, MD 21205, USA; b Department of Medicine, Johns Hopkins University School of Medicine, Baltimore, MD, USA; c Division of Intramural Research, National Institute of Allergy and Infectious Diseases, Baltimore, MD, USA

**Keywords:** COVID-19, People who inject drugs, People who use drugs, Substance use, Vaccination, Disparities

## Abstract

**Background:**

SARS-CoV-2 serosurveys can help characterize disparities in SARS-CoV-2 infection and identify gaps in population immunity. Data on SARS-CoV-2 seroprevalence among people who inject drugs (PWID) are limited.

**Methods:**

We conducted a cross-sectional study between December 2020 and July 2022 among 561 participants in the AIDS Linked to the IntraVenous Experience (ALIVE) study—a community-based cohort of current and former PWID in Baltimore, Maryland. Serum samples were assayed for infection-induced anti-nucleocapsid (anti-N) and infection and/or vaccination-induced anti-spike-1 (anti-S) SARS-CoV-2 IgG. We estimated adjusted prevalence ratios (aPR) via modified Poisson regression models.

**Results:**

The median age was 59 years, 35% were female, 84% were non-Hispanic Black, and 16% reported recent injection drug use. Anti-N antibody prevalence was 26% and anti-S antibody prevalence was 63%. Anti-N and anti-S antibody prevalence increased over time. Being employed (aPR=1.53 [95%CI=1.11–2.11]) was associated with higher anti-N prevalence, while a cancer history (aPR=0.40 [95%CI=0.17–0.90]) was associated with lower anti-N prevalence. HIV infection was associated with higher anti-S prevalence (aPR=1.13 [95%CI=1.02–1.27]), while younger age and experiencing homelessness (aPR=0.78 [95%CI=0.60–0.99]) were factors associated with lower anti-S prevalence. Substance use-related behaviors were not significantly associated with anti-N or anti-S prevalence.

**Conclusions:**

SARS-CoV-2 seroprevalence increased over time among current and former PWID, suggesting cumulative increases in the incidence of SARS-CoV-2 infection and vaccination; however, there were disparities in infection-induced seroprevalence and infection and/or vaccine-induced seroprevalence within this study sample. Dedicated prevention and vaccination programs are needed to prevent disparities in infection and gaps in population immunity among PWID during emerging epidemics.

## Highlights

• Prevalence of infection-induced SARS-CoV-2 antibodies was 26%.
• Prevalence of infection and/or vaccination-induced SARS-CoV-2 antibodies was 63%.
• The prevalence and magnitude of SARS-CoV-2 antibodies increased over time.
• Substance use-related behaviors were not associated with SARS-CoV-2 antibodies.
• Disparities in seroprevalence highlighted groups that need greater vaccine efforts.

## 1 Introduction

The coronavirus disease 2019 (COVID-19) pandemic, caused by the severe acute respiratory syndrome coronavirus 2 (SARS-CoV-2) virus, has disproportionately impacted socially marginalized populations (Barron et al., 2022). People who inject drugs (PWID) face many socio-structural and economic inequities, some of which have been exacerbated by the COVID-19 pandemic (Bradley et al., 2022; Carroll et al., 2023; Genberg et al., 2021; Wiessing et al., 2023). Studies conducted in the first two years of the pandemic have found low engagement in infection prevention behaviors (e.g., physical distancing) (Genberg et al., 2021), limited uptake of COVID-19 testing (Gorbach et al., 2022; Yeager et al., 2022), and suboptimal coverage of COVID-19 vaccination among PWID (Cepeda et al., 2022; Cioffi et al., 2022; Iversen et al., 2022; Strathdee et al., 2023a). PWID also often have chronic comorbidities (Heidari et al., 2022; Lim et al., 2022; Sun et al., 2022), which are risk factors for developing severe COVID-19 outcomes (Ng et al., 2021). Data from medical records have found that people with substance use disorders have been associated with an increased risk of severe COVID-19 outcomes compared to people without substance use disorders (Ao et al., 2022; Hasin et al., 2022; Krawczyk et al., 2023; Wang et al., 2021). Nonetheless, there are limited population-based data on the epidemiology of SARS-CoV-2 infection among people who use drugs, including PWID.

Case-based surveillance approaches to evaluate SARS-CoV-2 infection risk are inadequate because they under-ascertain COVID-19 cases, do not capture asymptomatic SARS-CoV-2 infections, and are susceptible to biases associated with heterogeneities in COVID-19 testing and reporting (Accorsi et al., 2021; Alwan, 2020; Oran and Topol, 2021). Alternatively, population-based serosurveys that measure antibodies to SARS-CoV-2 can provide insight into the cumulative incidence of infection and the degree of population-level humoral immunity induced by prior infection and/or vaccination in a population (Bergeri et al., 2022; Clapham et al., 2020). In settings like the United States that have rolled out COVID-19 vaccines that target the spike protein of SARS-CoV-2, detection of immunoglobulin-G (IgG) to the nucleocapsid protein (anti-N) of SARS-CoV-2 is an indicator of prior (or recent) exposure to the virus (i.e., infection) and detection of IgG to the spike protein (anti-S) of SARS-CoV-2 is an indicator of prior (or recent) infection and/or vaccination. These data can help identify risk factors of SARS-CoV-2 infection and characterize susceptible populations that may be at risk of severe COVID-19 outcomes due to limited immunity (e.g., no prior exposure to SARS-CoV-2 by infection and/or vaccination). This information can inform which populations require increased prevention efforts including COVID-19 vaccination.

There are sparse data on infection- and vaccination-induced SARS-CoV-2 seroprevalence among PWID. In a study conducted in the early pandemic period between June 2020 and October 2020, SARS-CoV-2 seroprevalence was 5.4% among clients of needle exchange programs in Stockholm, Sweden (Lindqvist et al., 2021). Prior to the introduction of the B.1.617.2 (Delta) and B.1.1.529 (Omicron) variants of SARS-CoV-2 and widespread availability of COVID-19 vaccines, a community-based study of people currently injecting drugs that was conducted between October 2020 and June 2021 in San Diego, California and Tijuana, Mexico found that the SARS-CoV-2 seroprevalence was 36%, which exceeded estimates from the general population in each city at that time (Strathdee et al., 2021). In a mixed sample of PWID and their sexual or injecting partners recruited at needle and syringe service programs and methadone clinics in Kenya between April 2021 and July 2021, 31% had anti-N antibodies (Doshi et al., 2023). Given that the spread of SARS-CoV-2 has been heterogeneous by population, geography, and time throughout the pandemic, these findings on infection risk may not be generalizable or transportable to other populations, settings, or points in time (Rudolph et al., 2023). Previous studies have highlighted disparities in COVID-19 vaccine uptake based on self-reported and medical record data among current and former PWID (Cepeda et al., 2022; Des Jarlais et al., 2023; Iversen et al., 2022; Strathdee et al., 2023a); however, there remains limited data characterizing the distribution of infection and/or vaccination-induced anti-S antibodies among PWID following widespread SARS-CoV-2 infection and vaccination.

In this cross-sectional study, we characterize the prevalence and correlates of anti-N and anti-S antibodies in a community-recruited sample of current and former PWID residing in or near Baltimore, Maryland.

## 2 Methods

### 2.1 Study population and procedures

The AIDS Linked to the IntraVenous Experience (ALIVE) study is a prospective community-recruited cohort of current and former PWID, located in Baltimore, Maryland (Vlahov et al., 1991). Since 1988, there have been 5506 participants enrolled over five recruitment periods. Eligible participants were ≥18 years of age and reported a history of injection drug use. Before the COVID-19 pandemic, participants attended semi-annual in-person visits at a research clinic where they completed interviewer-administered and self-administered surveys, provided a blood specimen, and underwent a clinical exam. Due to the pandemic, in-person semi-annual visits were suspended on March 13, 2020. The study transitioned to collecting survey data for routine semi-annual visits by telephone in December 2020. The research clinic also re-opened in December 2020, and blood specimen collection resumed in-person with the implementation of COVID-19 mitigation procedures (e.g., indoor masking). Serum samples were stored at -80°C. Of note, study staff provide participants informational resources on health services including where and how to receive testing and vaccination for various infectious diseases. This study was approved by the Johns Hopkins Bloomberg School of Public Health Institutional Review Board. Participants provided written informed consent for in-person visits and oral consent for telephone surveys.

Of the 1241 participants who attended a semi-annual study visit between January 2018-February 2020 (i.e., just before the pandemic), 550 participants completed at least one semi-annual visit with blood specimen collection between December 15, 2020-July 26, 2022 (i.e., during the pandemic). Another 11 participants who were in follow-up before 2018 also had at least one semi-annual visit with blood specimen collection during the pandemic. This cross-sectional study used data from all participants’ first semi-annual visit with blood specimen collection during the pandemic (*n* = 561).

Compared to participants who were in follow-up just before the pandemic but did not complete a visit with blood specimen collection during the pandemic (*n* = 691), participants included in the analytic sample (*n* = 561) were older (median age [IQR], 58 [52–63] vs. 56 [48–61] years), more likely to identify as Black (85% vs. 73%), less likely to have attained a high school education or GED (44% vs. 49%), and more likely be living with HIV (36% vs. 24%) at their last pre-pandemic visit (Supplemental Table 1).

### 2.2 Survey measures

We obtained self-reported data on date of birth, sex, race and ethnicity and educational attainment from an in-person survey conducted at enrollment. All other self-reported data were obtained from a telephone survey corresponding with the time of sample collection, which collected data on additional demographic factors (i.e., marital status and cohabitating persons), socio-structural factors (i.e., experienced homelessness in the past 6 months, employment status, personal income (before taxes) in the past 6 months, and experienced incarceration in the past 6 months), behavioral health factors (e.g., hazardous alcohol use [AUDIT-C] and behaviors in the past 6 months including cocaine use, heroin use, fentanyl use by itself, injection drug use, syringe service program (SSP) attendance, prescription for methadone or buprenorphine, and engagement in transactional sex), and clinical factors (i.e., a history of ever being diagnosed with diabetes, hypertension, cardiovascular diseases, liver disease, cancer, and pulmonary disease). Hazardous alcohol use was defined by an AUDIT-C score ≥4 for males and ≥3 for females (Bradley et al., 2007; Bush et al., 1998). People living with HIV were also asked about antiretroviral therapy use in the past 6 months.

### 2.3 COVID-19 vaccination data

We manually extracted data on the dates of COVID-19 vaccination through February 02, 2022 from participants’ medical records in the Chesapeake Regional Information System for Our Patients (CRISP) (Cepeda et al., 2022; CRISP, 2023). CRISP shares electronic medical record data across Maryland and includes vaccination data provided by the Maryland Department of Health's vaccine surveillance system. Of 561 participants, we successfully extracted data on 543 participants using a probabilistic matching algorithm based on name and date of birth. We created a binary variable for a history of receiving ≥1 COVID-19 vaccine dose before the date of sample collection.

### 2.4 Laboratory testing

Serum samples were assayed for antibodies to the SARS-CoV-2 nucleocapsid (N) and spike (S) proteins via the V-Plex SARS-CoV-2 Panel 2 (IgG) kit, which is a multiplexed immunoassay that uses electrochemiluminescence detection technology (MesoScale Diagnostics, Gaithersburg, MD). We followed the manufacturer's protocol, and antibody levels are reported in arbitrary units per milliliter (AU/mL). Detection of anti-N and anti-S was defined using manufacturer's cutoffs (5000 and 1960 AU/mL, respectively), which according to the manufacturer, correspond to a sensitivity of 93.8% and specificity of 100.0% for anti-N and a sensitivity of 98.3% and specificity of 99.5% for anti-S.

Serum samples were also assayed for HIV-1 antibodies using an enzyme-linked immunosorbent assay and confirmatory Western blot testing.

### 2.5 Statistical analysis

The primary outcomes included the prevalence of anti-N detection and the prevalence of anti-S detection. We estimated Clopper-Pearson 95% confidence intervals (CI) for prevalence estimates. We examined anti-N and anti-S prevalence by calendar period (i.e., December 2020 to May 2021, June 2021 to November 2021, and December 2021 to July 2022). These periods reflect the introduction of SARS-CoV-2 variants in the general U.S. population, with the B.1.617.2 (Delta) variant wave beginning in June 2021 and B.1.1.529 (Omicron) variant wave beginning in December 2021 (Wang et al., 2022). These periods also generally coincide with changes in the availability of COVID-19 vaccines in Maryland. Specifically, there was a phased approach to vaccine distribution for high-risk populations in Maryland beginning in December 2020, until vaccine eligibility expanded to all people aged ≥16 years beginning on April 27, 2021. The U.S. Food and Drug Administration authorized COVID-19 booster doses for all people aged ≥18 years on November 19, 2021. We also fit generalized additive models to estimate the probability of anti-N and anti-S detection over continuous calendar time. We also examined the magnitude of anti-N and anti-S antibody levels by calendar period using the non-parametric Cuzick's test for trend.

In addition to calendar period, we explored demographic, socio-structural, behavioral, and clinical correlates of anti-N and anti-S prevalence. We estimated crude prevalence ratios (PR) using Poisson regression models with robust standard errors. For each covariate of interest, we estimated adjusted prevalence ratios (aPR) with a multivariable model that included adjustment for calendar period, age, sex, and race and ethnicity. We included adjustment for calendar period since SARS-CoV-2 transmission dynamics (e.g., transmissibility of the dominant variant) and vaccine access were known to vary by time during the study period. Due to sample size restrictions, we did not conduct subgroup analyses stratified by calendar period. However, we performed two sensitivity analyses. To assess the impact of potential waning effects of anti-N antibodies on study inferences (Arkhipova-Jenkins et al., 2021), we examined correlates of anti-N restricted to data from the early period before the introduction of the Omicron variant (i.e., December 2020-November 2021). We also examined correlates of anti-S restricted to the period after general access to the COVID-19 vaccine (i.e., May 2021-July 2022).

## 3 Results

Of 561 participants, the median age was 59 years (range 28–77), 34.9% (*n* = 196) were female, 83.8% (*n* = 470) were non-Hispanic Black, 36.2% (*n* = 203) were living with HIV (97.0% [*n* = 193] of which reported antiretroviral therapy use in the past 6 months), 16.0% (*n* = 90) reported recent injection drug use in the past 6 months, and 53.3% (*n* = 299) had documentation for previously receiving ≥1 COVID-19 vaccine dose (before sample collection) (Table 1). Characteristics of the study sample varied by calendar period. For instance, the percentage of participants with a documented record of ≥1 COVID-19 vaccination was 23.6% (*n* = 52) in December 2020-May 2021, 72.3% (*n* = 141) in June 2021-November 2021, and 72.6% (*n* = 106) in December 2021-July 2022.

**Table 1:** Characteristics of the study sample overall and by calendar period.

	No. Participants (%)			
	Total	Dec.2020-May.2021 (Pre-Delta)	June.2021-Nov.2021 (Delta wave)	Dec.2021-July.2022 (Omicron wave)
	N=561	N=220	N=195	N=146
Enrollment cohort				
Before 2005	221 (39.4)	84 (38.2)	77 (39.5)	60 (41.1)
2005-2008	156 (27.8)	71 (32.3)	50 (25.6)	35 (24.0)
2015-2018	184 (32.8)	65 (29.5)	68 (34.9)	51 (34.9)
Age group, y				
18-44	51 (9.1)	12 (5.5)	17 (8.7)	22 (15.1)
45-54	117 (20.9)	46 (20.9)	42 (21.5)	29 (19.9)
55-64	265 (47.2)	112 (50.9)	87 (44.6)	66 (45.2)
≥65	128 (22.8)	50 (22.7)	49 (25.1)	29 (19.9)
Sex				
Male	365 (65.1)	147 (66.8)	128 (65.6)	90 (61.6)
Female	196 (34.9)	73 (33.2)	67 (34.4)	56 (38.4)
Race and ethnicity				
Non-Hispanic Black	470 (83.8)	192 (87.3)	167 (85.6)	111 (76.0)
Non-Hispanic white	69 (12.3)	24 (10.9)	18 (9.2)	27 (18.5)
Other race and ethnicity	22 (3.9)	4 (1.8)	10 (5.1)	8 (5.5)
Educational attainment				
Less than H.S.	312 (55.6)	127 (57.7)	101 (51.8)	84 (57.5)
Completed H.S./GED or more	248 (44.2)	93 (42.3)	93 (47.7)	62 (42.5)
Marital status				
Never married	283 (50.4)	110 (50.0)	97 (49.7)	76 (52.1)
Widowed/divorced	137 (24.4)	56 (25.5)	48 (24.6)	33 (22.6)
Married	141 (25.1)	54 (24.5)	50 (25.6)	37 (25.3)
Lives alone^a^				
No	315 (56.1)	112 (50.9)	114 (58.5)	89 (61.0)
Yes	244 (43.5)	108 (49.1)	81 (41.5)	55 (37.7)
Experienced homelessness (6 mo.)				
No	500 (89.1)	206 (93.6)	167 (85.6)	127 (87.0)
Yes	61 (10.9)	14 (6.4)	28 (14.4)	19 (13.0)
Currently employed				
No	483 (86.1)	187 (85.0)	172 (88.2)	124 (84.9)
Yes	78 (13.9)	33 (15.0)	23 (11.8)	22 (15.1)
Personal income (6 mo.)^a^				
≥$5,000	142 (25.3)	53 (24.1)	48 (24.6)	41 (28.1)
<$5,000	407 (72.5)	164 (74.5)	144 (73.8)	99 (67.8)
Experienced incarceration (6 mo.)				
No	549 (97.9)	213 (96.8)	192 (98.5)	144 (98.6)
Yes	12 (2.1)	7 (3.2)	3 (1.5)	2 (1.4)
Transactional sex (6 mo.)				
No	545 (97.1)	216 (98.2)	187 (95.9)	142 (97.3)
Yes	16 (2.9)	4 (1.8)	8 (4.1)	4 (2.7)
Hazardous alcohol use (AUDIT-C)				
No	470 (83.8)	192 (87.3)	165 (84.6)	113 (77.4)
Yes	91 (16.2)	28 (12.7)	30 (15.4)	33 (22.6)
Cocaine use (6 mo.)				
No	404 (72.0)	174 (79.1)	134 (68.7)	96 (65.8)
Yes	157 (28.0)	46 (20.9)	61 (31.3)	50 (34.2)
Heroin or fentanyl use (6 mo.)				
No	384 (68.4)	168 (76.4)	130 (66.7)	86 (58.9)
Yes	177 (31.6)	52 (23.6)	65 (33.3)	60 (41.1)
Injection drug use (6 mo.)				
No	471 (84.0)	197 (89.5)	165 (84.6)	109 (74.7)
Yes	90 (16.0)	23 (10.5)	30 (15.4)	37 (25.3)
Attended SSP (6 mo.)^a^				
No	507 (90.4)	206 (93.6)	177 (90.8)	124 (84.9)
Yes	53 (9.4)	13 (5.9)	18 (9.2)	22 (15.1)
Prescribed MOUD (6 mo.)				
No	285 (50.8)	104 (47.3)	104 (53.3)	77 (52.7)
Yes	276 (49.2)	116 (52.7)	91 (46.7)	69 (47.3)
HIV status				
Living without HIV	358 (63.8)	122 (55.5)	130 (66.7)	106 (72.6)
Living with HIV	203 (36.2)	98 (44.5)	65 (33.3)	40 (27.4)
History of diabetes^a^				
No	430 (76.6)	164 (74.5)	154 (79.0)	112 (76.7)
Yes	129 (23.0)	54 (24.5)	41 (21.0)	34 (23.3)
History of hypertension^a^				
No	214 (38.1)	74 (33.6)	81 (41.5)	59 (40.4)
Yes	346 (61.7)	145 (65.9)	114 (58.5)	87 (59.6)
History of cardiovascular disease^a^				
No	438 (78.1)	164 (74.5)	161 (82.6)	113 (77.4)
Yes	119 (21.2)	53 (24.1)	33 (16.9)	33 (22.6)
History of liver disease^a^				
No	473 (84.3)	185 (84.1)	168 (86.2)	120 (82.2)
Yes	84 (15.0)	33 (15.0)	27 (13.8)	24 (16.4)
History of cancer^a^				
No	513 (91.4)	202 (91.8)	178 (91.3)	133 (91.1)
Yes	44 (7.8)	14 (6.4)	17 (8.7)	13 (8.9)
History of pulmonary disease^a^				
No	404 (72.0)	158 (71.8)	141 (72.3)	105 (71.9)
Yes	154 (27.5)	61 (27.7)	53 (27.2)	40 (27.4)
Received ≥1 COVID-19 dose				
No	244 (43.5)	167 (75.9)	44 (22.6)	33 (22.6)
Yes	299 (53.3)	52 (23.6)	141 (72.3)	106 (72.6)
*Unknown*	18 (3.2)	1 (0.5)	10 (5.1)	7 (4.8)

a Column percentages may not add to 100% due to missing data (<2.5% for each variable overall). Abbreviations: HIV, human immunodeficiency virus; MOUD, medications for opioid use disorder; SSP, syringe service programs.

Overall, anti-N antibody prevalence was 26.0% (*n* = 146 [95%CI=22.4–30.0]), anti-S antibody prevalence was 62.7% (*n* = 352 [95%CI=58.6–66.8]), and the prevalence of both types of antibodies was 23.9% (*n* = 134 [95%CI=20.4–27.6]). Among unvaccinated participants (*n* = 244), anti-N antibody prevalence was 23.4% (*n* = 57 [95%CI=18.2–29.2]) and anti-S antibody prevalence was 27.9% (*n* = 68 [95%CI=22.3–33.9]).

**Fig. 1:**
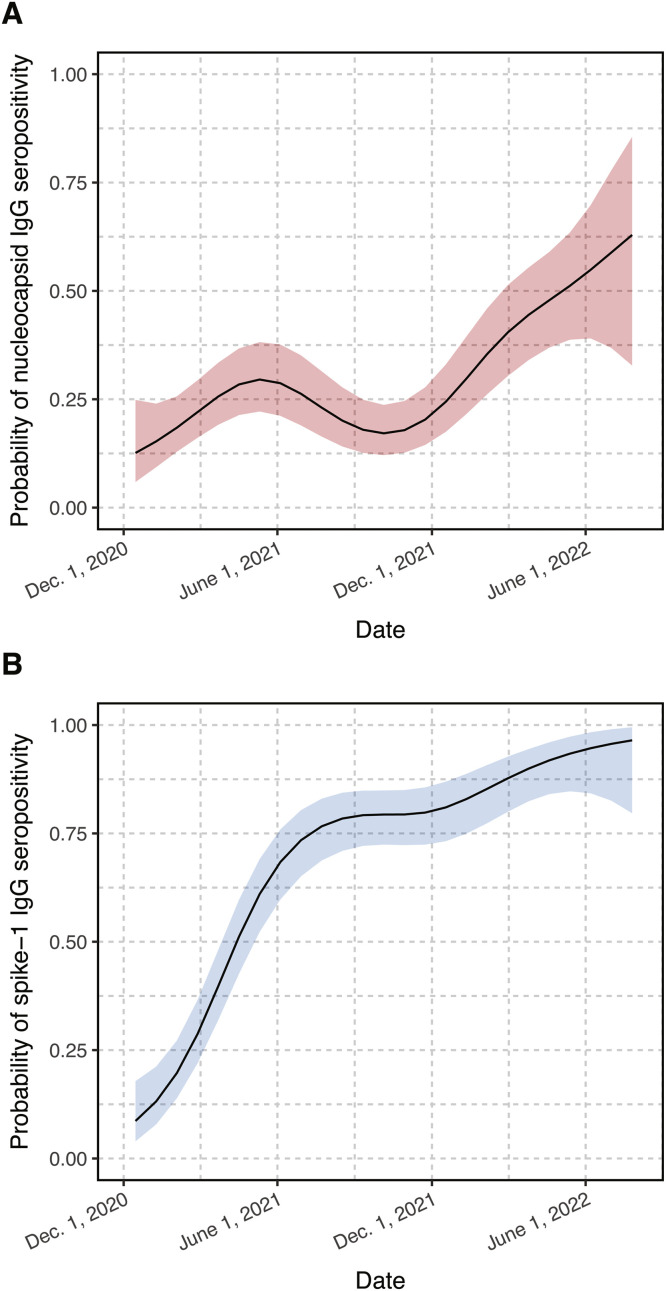
Probability of anti-*nucleocapsid* and anti-*spike-1* SARS-CoV-2 IgG seropositivity by calendar time. Each panel depicts probability estimates from generalized additive models with shaded areas indicating 95% confidence intervals.

There was a non-linear increase in the probability of anti-N antibody detection over the study period (December 2020 to July 2022), though there was a temporary decrease in the probability of anti-N antibody detection in the second half of 2021 (Fig. 1A). The probability of anti-S antibody detection increased over the study period (Fig. 1B). Between December 2020-May 2021 and December 2021-July 2022, the prevalence of anti-N antibodies significantly increased from 23.2% to 39.7% (PR=1.71 [95%CI=1.25–2.34]) and the prevalence of anti-S antibodies significantly increased from 33.6% to 85.6% (PR=2.55 [95%CI=2.09–3.10]) (Table 2). These associations were similar after adjustment for age, sex, and race and ethnicity. The magnitude of anti-N and anti-S antibody levels followed similar trends and significantly increased over time between December 2020-May 2021 and December 2021-July 2022 (p-trend, *p* = 0.002 and *p* < 0.001, respectively; Supplemental Fig. 1).

**Table 2:** Trends in anti-SARS-CoV-2 IgG seroprevalence by calendar period.

Calendar period	N	anti-Nucleocapsid IgG seroprevalence			anti-Spike-1 IgG seroprevalence		
		n (%)	PR (95% CI)	aPR (95% CI)⁎	n (%)	PR (95% CI)	aPR (95% CI)⁎
Dec. 2020-May 2021	220	51 (23.2)	Ref.	Ref.	74 (33.6)	Ref.	Ref.
June 2021-Nov. 2021	195	37 (19.0)	0.82 (0.56-1.19)	0.82 (0.56-1.19)	153 (78.5)	2.33 (1.91-2.85)	2.34 (1.92-2.85)
Dec. 2021-July 2022	146	58 (39.7)	1.71 (1.25-2.34)	1.78 (1.30-2.43)	125 (85.6)	2.55 (2.09-3.10)	2.63 (2.17-3.20)

⁎ Adjusted prevalence ratios (aPR) were estimated by multivariable modified Poisson regression with robust variance estimation that included adjustment for age, sex, and race and ethnicity. Abbreviations: aPR, adjusted prevalence ratio, CI, confidence interval PR, prevalence ratio.

**Fig. 2:**
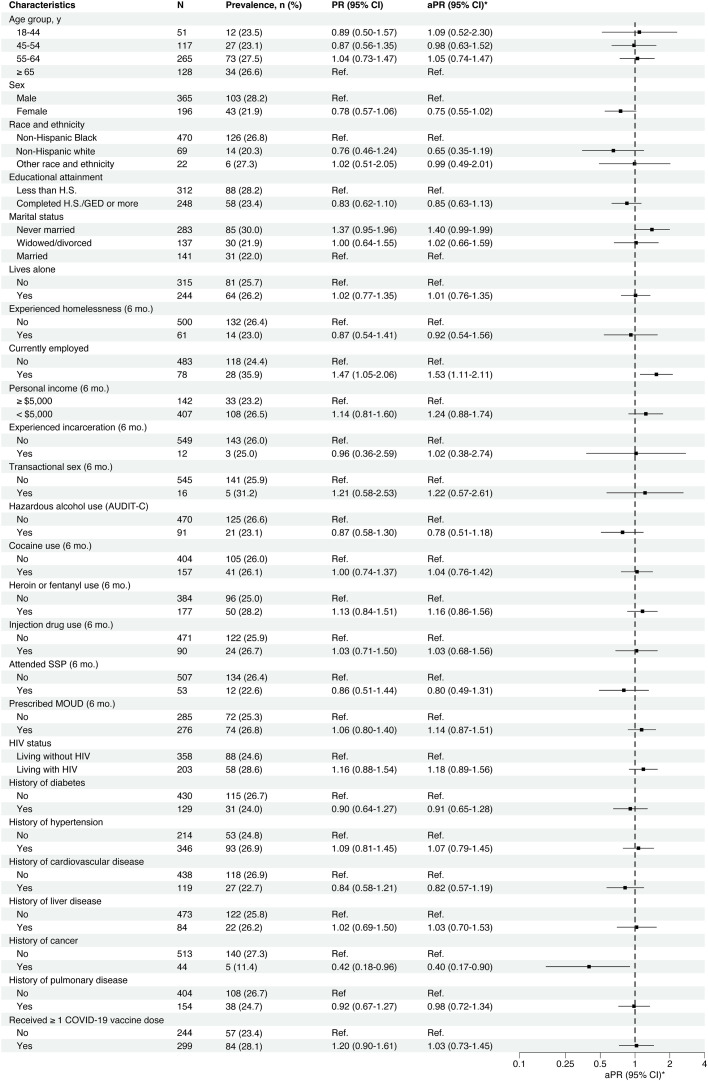
Correlates of anti-*nucleocapsid* SARS-CoV-2 IgG seroprevalence. * Adjusted prevalence ratios (aPR) were estimated by Poisson regression with robust variance estimation; a separate model was used for each covariate shown and all models included adjustment for calendar period, age, sex, and race and ethnicity.

Age and race and ethnicity were not significantly associated with the prevalence of anti-N antibodies in crude analyses or the multivariable model including calendar period, age, sex, and race and ethnicity (Fig. 2). Compared to male participants, the prevalence of anti-N antibodies was lower among female participants in the crude analysis (PR=0.78 [95%CI=0.57–1.06]) and the adjusted analysis (aPR=0.75 [95%CI=0.55–1.02]), but neither estimate of association was statistically significant. Similarly, compared to married participants, the prevalence of anti-N antibodies was higher among never married participants in the crude analysis (PR=1.37 [95%CI=0.95–1.96]) and adjusted analysis (aPR=1.40 [95%CI=0.99–1.99]), but neither estimate of association was statistically significant. Compared to unemployed participants, employed participants had a significantly higher prevalence of anti-N antibodies in the crude analysis (PR=1.47 [95%CI=1.05–2.06]) and adjusted analysis (aPR=1.53 [95%CI=1.11–2.11]). A cancer history was significantly associated with a lower prevalence of anti-N antibodies in the crude analysis (PR=0.42 [95%CI=0.18–0.96]) and adjusted analysis (aPR=0.40 [95%CI=0.17–0.90]. Other comorbidities and substance use-related behaviors were not significantly associated with the prevalence of anti-N antibodies.

**Fig. 3:**
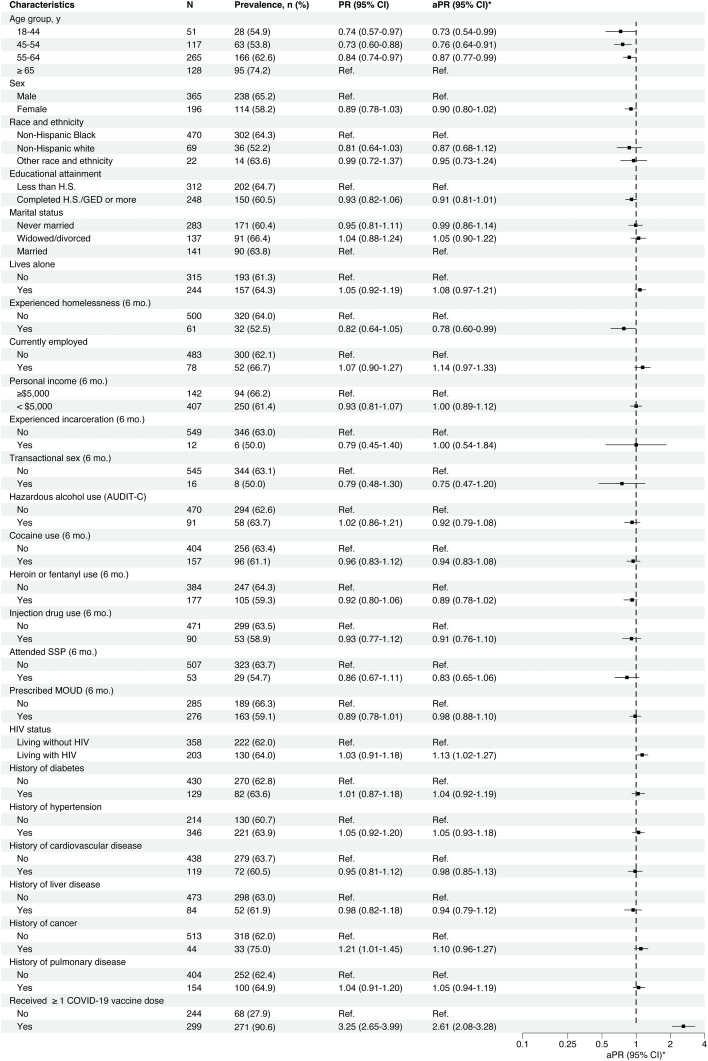
Correlates of anti-*spike-1* SARS-CoV-2 IgG seroprevalence. * Adjusted prevalence ratios (aPR) were estimated by Poisson regression with robust variance estimation; a separate model was used for each covariate shown and all models included adjustment for calendar period, age, sex, and race and ethnicity.

Younger age was significantly associated with a lower prevalence of anti-S antibodies in crude and adjusted analyses (Fig. 3). Compared to participants aged ≥65 years, the prevalence of anti-S antibodies was significantly lower among participants aged 55–64 years (aPR=0.87 [95%CI=0.77–0.99]), participants aged 45–54 years (aPR=0.76 [95%CI=0.64–0.91]), and those aged 18–44 years (aPR=0.73 [95%CI=0.54–0.99]). Living with HIV (aPR=1.13 [95%CI=1.02–1.27]) and having recently experienced homelessness (aPR=0.78 [95%CI=0.60–0.99]) were additional factors significantly associated with anti-S antibodies in adjusted analyses. A history of cancer was significantly associated with a higher prevalence of anti-S antibodies in the crude analysis (PR=1.21 [95%CI=1.01–1.45]), but this association was attenuated and not statistically significant in the adjusted analysis (aPR=1.10 [95%CI=0.96–1.27]). The prevalence of anti-S antibodies varied by other factors, but these differences were not statistically significant in crude or adjusted analyses. For instance, compared to male participants, the prevalence of anti-S antibodies was marginally lower among female participants in the crude analysis (PR=0.89 [95%CI=0.78–1.03]) and adjusted analysis (aPR=0.90 [95%CI=0.80–1.02]), but neither estimate of association was statistically significant.

Having received ≥1 dose of a COVID-19 vaccine was significantly associated with a higher prevalence of anti-S antibodies in the crude analysis (PR=3.25 [95%CI=2.65–3.99]) and adjusted analysis (aPR=2.61 [95%CI=2.08–3.28]) (Fig. 3).

Associations with anti-N antibodies were generally in the same direction as the primary analysis when restricting the analysis to the early periods (December 2020-November 2021) (*n* = 415) (Supplemental Table 2). Associations with anti-S antibodies were also largely in the same direction as the primary analysis when restricted to the period after general access to the vaccine (May 2021-July 2022) (*n* = 381) (Supplemental Table 3).

## 4 Discussion

In this community-recruited sample of current and former PWID in Baltimore, Maryland, the prevalence and magnitude of anti-N and anti-S antibodies increased over time with greater increases in anti-S antibody levels, suggesting cumulative increases in SARS-CoV-2 incidence of infection and vaccination in this population. However, there were disparities in anti-N antibody prevalence and anti-S antibody prevalence, suggesting there was heterogeneity in both SARS-CoV-2 incidence and vaccination in this sample of current and former PWID. For example, employed participants were more likely to have infection-induced anti-N antibodies, and younger participants were less likely to have infection and/or vaccination-induced anti-S antibodies. Serosurveillance can be a tool to identify populations that are at heightened risk of COVID-19 and face barriers to key interventions even within a vulnerable population.

The seroprevalence of SARS-CoV-2 in the general population of Baltimore and Maryland has not been characterized. The prevalence of anti-N (39.7%) and anti-S (85.6%) antibodies during December 2021-July 2022 in this sample of current and former PWID is fairly consistent with preliminary estimates of anti-N (41.6%) and anti-S (91.5%) antibody prevalence among a national sample of adults in the U.S. household population during August 2021-May 2022 (Akinbami et al., 2022). However, given that older age is strongly associated with a lower prevalence of anti-N antibodies in the U.S., and our study sample was much older than the adults in the U.S. household population, this sample may have had a higher prevalence of anti-N antibodies than the U.S. household population after accounting for age. It is notable that the prevalence of anti-N antibodies in this sample during December 2020-May 2021 (23.2%) was lower than the SARS-CoV-2 seroprevalence estimate among community-recruited PWID in San Diego and Tijuana (36.3%) during October 2020-June 2021 (Strathdee et al., 2021). This may also be due to a differential age distribution between samples, and differences in the prevalence of behavioral and socio-structural risk factors of SARS-CoV-2 infection (e.g., sex work and incarceration). It could also be due to geographic differences in the amount of community spread of SARS-CoV-2.

The seroprevalence of SARS-CoV-2 is expected to increase over time given it reflects the cumulative incidence of infection and vaccination. We observed notable increases in the prevalence and magnitude of anti-N and anti-S antibodies over the study period. The observed increase in anti-N antibody levels in this study after December 2021 is consistent with the introduction of the more transmissible Omicron variant of SARS-CoV-2 at the end of December 2021 and parallels observed increases in the prevalence of anti-N antibodies in other US populations (Jones et al., 2021; Wiegand et al., 2023). Similarly, the increase in the prevalence of anti-S antibodies in this study is consistent with increases in the uptake of COVID-19 vaccination in this sample and in the general U.S. adult population over time (Kriss et al., 2022). Even though we adjusted for demographic characteristics, the observed changes over time may also partly be due to differences in participant characteristics over time, given the cross-sectional study design. Longitudinal studies are needed to monitor SARS-CoV-2 infection risk and population humoral immunity in vulnerable populations, including PWID.

We identified different correlates of infection-induced antibodies than the previous community-based study of PWID in San Diego and Tijuana (Strathdee et al., 2021). While being employed was significantly associated with a greater prevalence of anti-N antibodies, and male participants and those who were never married had a marginally higher prevalence of anti-N antibodies, the prior study did not find these differences. The association of employment and prevalence of anti-N antibodies may be explained by occupational risks or an inability to adhere to physical distancing. Although the prior study found positive associations between incarceration and engagement in sex work and infection-induced SARS-CoV-2 seroprevalence, our study was underpowered to detect these associations. It is notable that our study and two prior studies did not find associations between recent substance use behaviors and infection-induced SARS-CoV-2 seroprevalence (Doshi et al., 2023; Strathdee et al., 2021). While substance use may play a role in the pathogenesis of COVID-19 (Ao et al., 2022; Hasin et al., 2022; Krawczyk et al., 2023; Wang et al., 2021), it may not be a salient risk factor of SARS-CoV-2 infection among people who use drugs. Research is also needed to understand the impact of macro-level factors (e.g., socio-structural inequities) on SARS-CoV-2 infection risk among PWID (Bradley et al., 2022).

There was heterogeneity in the prevalence of anti-S antibodies. The finding that anti-S antibody prevalence was significantly lower among younger participants and marginally lower in female participants is consistent with our prior work in this cohort that found younger and female participants were less likely to be vaccinated against COVID-19 during March to June 2021 (Cepeda et al., 2022). The association of age and sex with the probability of COVID-19 vaccine uptake has varied in other samples of PWID (Des Jarlais et al., 2023; Harvey-Vera et al., 2022; Iversen et al., 2022; Strathdee et al., 2023a). Notably, in our cohort, we also previously found that younger and female participants were also more likely to express high levels of COVID-19 vaccine hesitancy (Cepeda et al., 2022). Even in New York City where there were many efforts to improve COVID-19 vaccination coverage among vulnerable populations which facilitated high vaccine uptake among PWID (81%), the strongest predictor of remaining unvaccinated was having negative attitudes about the vaccine (Des Jarlais et al., 2023). PWID who remain unvaccinated may be misinformed about the COVID-19 vaccine, have institutional and/or medical distrust, and/or a low perceived risk of COVID-19 (Aronson et al., 2022; Valasek et al., 2022). We also found that persons who recently experienced homelessness were less likely to have detectable anti-S antibodies. In addition to the aforementioned reasons, this may also be reflective of social and structural barriers to COVID-19 vaccination. Regardless, our data highlight that programs and policies are needed to enhance COVID-19 vaccination coverage among current and former PWID to achieve equitable levels of population-level humoral immunity in this population (Strathdee et al., 2023b).

Participants with HIV had a higher prevalence of anti-S antibodies after adjustment for demographic factors, consistent with prior research demonstrating positive associations between HIV serostatus and COVID-19 vaccine uptake in this cohort and other samples of PWID (Cepeda et al., 2022; Iversen et al., 2022; Strathdee et al., 2023a). Participants with HIV in the current study sample were highly engaged in HIV care (e.g., 97% reported antiretroviral therapy use), which may have facilitated more encouragement and/or opportunity to receive the vaccine. People with HIV may also have a greater perceived need for the vaccine due to concerns about their potential immunocompromised status. Interestingly, a cancer history was negatively associated with the prevalence of anti-N antibodies and positively associated with the prevalence of anti-S antibodies. For similar reasons, participants with a cancer history may have been more likely to engage in infection prevention behaviors (e.g., physical distancing) and receive the COVID-19 vaccine. It was recently reported that having a greater number of chronic illnesses was associated with a higher incidence of COVID-19 vaccination among PWID in San Diego (Strathdee et al., 2023a).

Although this community-based study comprehensively characterizes the seroprevalence of SARS-CoV-2 in a large sample of a vulnerable population, it has limitations. We were unable to establish temporality between reported associations given the cross-sectional study design and were underpowered to detect some associations (e.g., transactional sex). In addition, there may be error in the measurement of self-reported variables (e.g., recall and social desirability bias) and anti-SARS-CoV-2 antibodies. While the study used a sensitive quantitative assay with a broad dynamic range to measure anti-SARS-CoV-2 IgG, a small fraction of individuals do not seroconvert following infection (i.e., develop detectable SARS-CoV-2 antibodies) and anti-SARS-CoV-2 IgG levels have been shown to wane over time (Arkhipova-Jenkins et al., 2021; Holmer et al., 2023; Stone et al., 2022). The slightly lower prevalence of anti-N (23.4%) as compared to anti-S (27.9%) among unvaccinated participants is consistent with the lower sensitivity of the assay to detect anti-N antibodies than anti-S antibodies, and that anti-N IgG levels have been shown to wane more quickly than anti-S IgG levels. Indeed, the observed decline in the prevalence of anti-N antibodies between June 2021 and November 2021 may be partly due to waning antibody levels and/or differences in sample characteristics over time. Importantly, the study may have underestimated the cumulative incidence of SARS-CoV-2 infection. It is reassuring, however, that correlates of anti-N antibodies restricted to the early pre-Omicron period were similar to the main analysis. Finally, while it is a strength that this study was conducted among an underrepresented population of older, predominantly Black sample of current and former PWID with a high prevalence of HIV and MOUD use, there was underrepresentation of certain groups including young people, people experiencing homelessness, and people currently injecting drugs. Thus, the study results may have limited generalizability to the broader population of current and former PWID in Baltimore and limited transportability to other populations of people who use drugs, especially beyond this urban setting.

In summary, we observed dynamic changes in the seroepidemiology of SARS-CoV-2 among a community-based sample of people with a history of injection drug use. We report disparities in infection-induced seroprevalence and infection- and/or vaccine-induced seroprevalence, highlighting populations that may require increased programmatic efforts to improve COVID-19 vaccination coverage. More broadly, these data indicate dedicated policies and programs are needed to ensure equitable access to and uptake of vaccination within vulnerable populations during emerging epidemics. Continued serosurveillance of SARS-CoV-2 among PWID is needed to monitor the degree of population-level humoral immunity in this vulnerable population.

## Funding source

This study was supported by the Division of Intramural Research, National Institute of Allergy and Infectious Diseases (NIAID), and by extramural support from NIAID (T32AI102623 [EUP]) and the National Institute on Drug Abuse (NIDA) (F31DA054849 [EUP], R01DA053136 [BLG], and U01DA036297 [GDK, SHM]). The study was also supported by the Johns Hopkins University Center for AIDS Research (CFAR), an NIH funded program (10P30AI094189), which is supported by the following NIH Institutes: NIAID, NCI, NICHD, NHLBI, NIDA, NIA, NIGMS, NIDDK, NIMHD.

## Role of funding source

The funders of the study had no role in study design, data collection, data analysis, data interpretation, writing of the report, or the decision to submit for publication; thus, the content of this manuscript is solely the responsibility of the authors and does not necessarily represent the views of the funding organizations.

## Previous presentation

This work was presented in part at the *Conference on Retroviruses and Opportunistic Infections*, February 19 to February 22, 2023, Seattle, Washington, USA.

## CRediT authorship contribution statement

**Eshan U. Patel:** Conceptualization, Formal analysis, Methodology, Visualization, Writing – original draft. **Shruti H. Mehta:** Conceptualization, Data curation, Supervision, Funding acquisition, Methodology, Writing – review & editing. **Becky L. Genberg:** Data curation, Supervision, Writing – review & editing. **Owen R. Baker:** Investigation, Data curation, Writing – review & editing. **Catherine G. Schluth:** Data curation, Investigation, Writing – review & editing. **Jacquie Astemborski:** Data curation, Investigation, Writing – review & editing. **Reinaldo E. Fernandez:** Investigation, Writing – review & editing. **Thomas C. Quinn:** Funding acquisition, Writing – review & editing. **Gregory D. Kirk:** Funding acquisition, Methodology, Writing – review & editing. **Oliver Laeyendecker:** Conceptualization, Data curation, Supervision, Writing – review & editing.

## Declaration of Competing Interest

No conflict declared.

## Appendix Supplementary materials

Image, application 1
