# Supplementary material for: Prevalence and correlates of SARS-CoV-2 seropositivity among people who inject drugs in Baltimore, Maryland
Source: Drug Alcohol Depend Rep. 2023 Aug 9;8:100184. doi: 10.1016/j.dadr.2023.100184 (PMC10450408; doi:10.1016/j.dadr.2023.100184)
Supplement: Supplementary file 1 [file mmc1.docx]

Supplemental Table 1. *Pre-pandemic* characteristics of participants in the analytic sample compared to those who were not in the analytic sample but were actively in follow-up in 2018-Feb 2020 (i.e., before the pandemic).

| Characteristics | Not included | Included | χ^2^  *p* value |
| --- | --- | --- | --- |
|  | N=691 | N=561 |  |
| Enrollment cohort |  |  | <0.001 |
| Before 2005 | 32% | 39% |  |
| 2005-2008 | 26% | 28% |  |
| 2015-2018 | 43% | 33% |  |
| Age group, y |  |  | <0.001 |
| 18-44 | 19% | 10% |  |
| 45-54 | 28% | 26% |  |
| 55-64 | 41% | 47% |  |
| ≥65 | 11% | 16% |  |
| Sex |  |  | 0.290 |
| Male | 68% | 65% |  |
| Female | 32% | 35% |  |
| Race |  |  | <0.001 |
| Black | 73% | 85% |  |
| Other race | 27% | 15% |  |
| Educational attainment |  |  | 0.075 |
| Less than H.S. | 51% | 56% |  |
| Completed H.S./GED or more | 49% | 44% |  |
| HIV status |  |  | <0.001 |
| Living without HIV | 76% | 64% |  |
| Living with HIV | 24% | 36% |  |

Supplemental Figure 1. Magnitude of anti-nucleocapsid and anti-spike-1 SARS-CoV-2 IgG levels by calendar period. Note: Dotted lines indicate the manufacturer’s threshold for seropositivity.


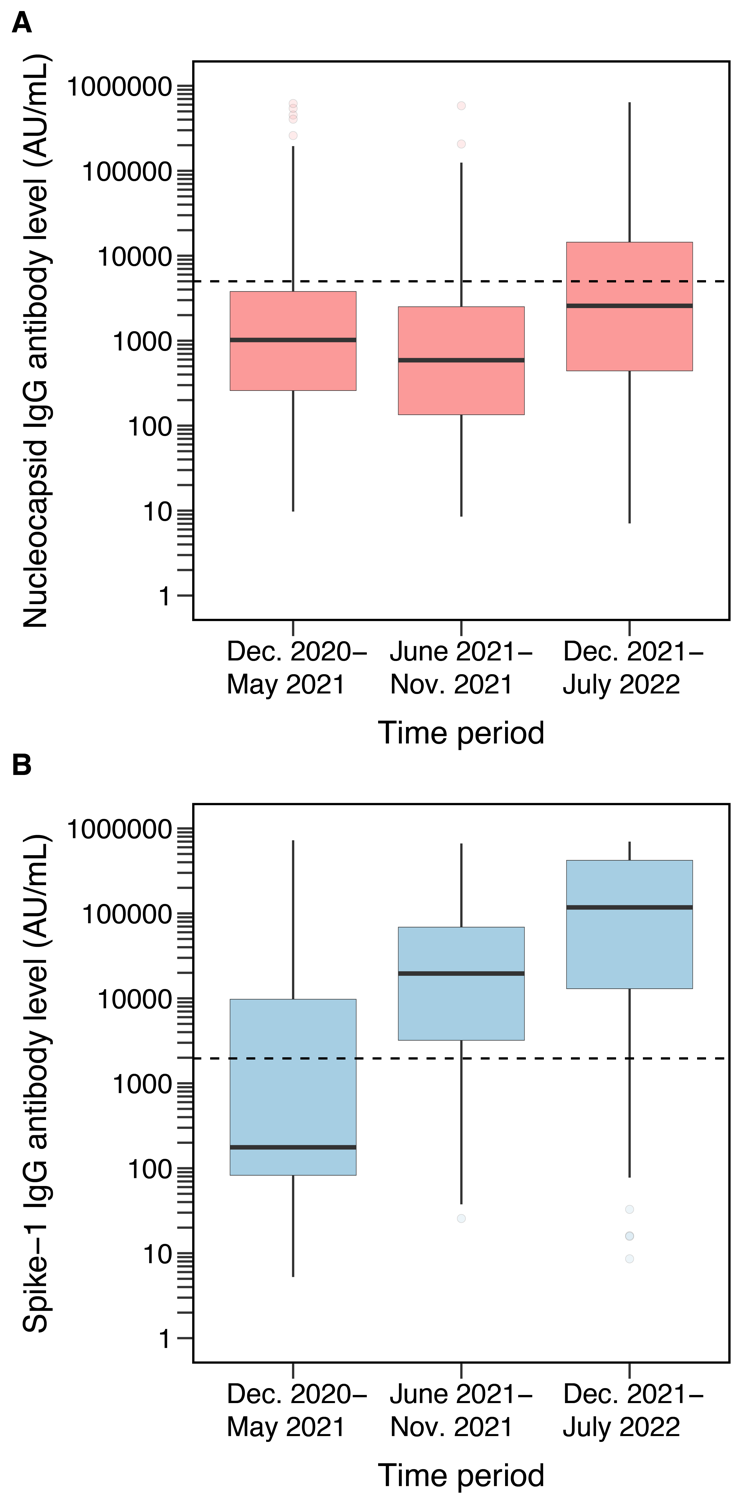


Supplemental Table 2. Sensitivity analysis – prevalence and correlates of anti-*nucleocapsid* SARS-CoV-2 IgG antibodies in the pre-Omicron era (n = 415).

|  | Total | Prevalence,  n (%) | Crude  Prevalence Ratio  (95% CI) | Adjusted  Prevalence Ratio*  (95% CI) |
| --- | --- | --- | --- | --- |
| Calendar period |  |  |  |  |
| Dec.2020-May.2021 | 220 | 51 (23.2) | Ref. | Ref. |
| June.2021-Nov.2021 | 195 | 37 (19.0) | 0.82 (0.56-1.19) | 0.83 (0.57-1.20) |
| Age group, y |  |  |  |  |
| 18-44 | 29 | 5 (17.2) | 0.78 (0.32-1.87) | 1.76 (0.55-5.58) |
| 45-54 | 88 | 16 (18.2) | 0.82 (0.46-1.46) | 1.07 (0.61-1.89) |
| 55-64 | 199 | 45 (22.6) | 1.02 (0.65-1.60) | 1.10 (0.71-1.72) |
| ≥65 | 99 | 22 (22.2) | Ref. | Ref. |
| Sex |  |  |  |  |
| Male | 275 | 67 (24.4) | Ref. | Ref. |
| Female | 140 | 21 (15.0) | 0.62 (0.39-0.96) | 0.60 (0.39-0.94) |
| Race and ethnicity |  |  |  |  |
| Non-Hispanic Black | 359 | 82 (22.8) | Ref. | Ref. |
| Non-Hispanic white | 42 | 5 (11.9) | 0.52 (0.22-1.21) | 0.38 (0.12-1.19) |
| Other including Hispanic | 14 | 1 (7.1) | 0.31 (0.05-2.09) | 0.30 (0.04-2.37) |
| Educational attainment |  |  |  |  |
| Less than H.S. | 228 | 53 (23.2) | Ref. | Ref. |
| Completed H.S./GED or more | 186 | 35 (18.8) | 0.81 (0.55-1.18) | 0.81 (0.55-1.18) |
| Marital status |  |  |  |  |
| Never married | 207 | 49 (23.7) | 1.37 (0.84-2.23) | 1.37 (0.85-2.21) |
| Widowed/divorced | 104 | 21 (20.2) | 1.17 (0.66-2.06) | 1.26 (0.71-2.24) |
| Married | 104 | 18 (17.3) | Ref. | Ref. |
| Lives alone |  |  |  |  |
| No | 226 | 47 (20.8) | Ref. | Ref. |
| Yes | 189 | 41 (21.7) | 1.04 (0.72-1.51) | 0.96 (0.65-1.43) |
| Experienced homelessness (6 mo.) |  |  |  |  |
| No | 373 | 81 (21.7) | Ref. | Ref. |
| Yes | 42 | 7 (16.7) | 0.77 (0.38-1.55) | 0.99 (0.46-2.17) |
| Currently employed |  |  |  |  |
| No | 359 | 73 (20.3) | Ref. | Ref. |
| Yes | 56 | 15 (26.8) | 1.32 (0.82-2.13) | 1.39 (0.87-2.22) |
| Personal income (6 mo.) |  |  |  |  |
| ≥$5,000 | 101 | 19 (18.8) | Ref. | Ref. |
| <$5,000 | 308 | 69 (22.4) | 1.19 (0.75-1.88) | 1.28 (0.81-2.03) |
| Experienced incarceration (6 mo.) |  |  |  |  |
| No | 405 | 85 (21.0) | Ref. | Ref. |
| Yes | 10 | 3 (30.0) | 1.43 (0.54-3.76) | 1.41 (0.59-3.38) |
| Transactional sex (6 mo.) |  |  |  |  |
| No | 403 | 85 (21.1) | Ref. | Ref. |
| Yes | 12 | 3 (25.0) | 1.19 (0.44-3.22) | 1.09 (0.38-3.08) |
| Hazardous alcohol use (AUDIT-C) |  |  |  |  |
| No | 357 | 75 (21.0) | Ref. | Ref. |
| Yes | 58 | 13 (22.4) | 1.07 (0.63-1.79) | 1.01 (0.59-1.73) |
| Cocaine use (6 mo.) |  |  |  |  |
| No | 308 | 68 (22.1) | Ref. | Ref. |
| Yes | 107 | 20 (18.7) | 0.85 (0.54-1.33) | 0.94 (0.59-1.51) |
| Heroin or fentanyl use (6 mo.) |  |  |  |  |
| No | 298 | 65 (21.8) | Ref. | Ref. |
| Yes | 117 | 23 (19.7) | 0.90 (0.59-1.38) | 0.99 (0.64-1.53) |
| Injection drug use (6 mo.) |  |  |  |  |
| No | 362 | 79 (21.8) | Ref. | Ref. |
| Yes | 53 | 9 (17.0) | 0.78 (0.42-1.46) | 0.85 (0.43-1.71) |
| Attended SSP (6 mo.) |  |  |  |  |
| No | 383 | 85 (22.2) | Ref. | Ref. |
| Yes | 31 | 3 (9.7) | 0.44 (0.15-1.30) | 0.42 (0.15-1.17) |
| Prescribed MOUD (6 mo.) |  |  |  |  |
| No | 208 | 43 (20.7) | Ref. | Ref. |
| Yes | 207 | 45 (21.7) | 1.05 (0.73-1.52) | 1.12 (0.78-1.60) |
| HIV status |  |  |  |  |
| Living without HIV | 252 | 48 (19.0) | Ref. | Ref. |
| Living with HIV | 163 | 40 (24.5) | 1.29 (0.89-1.87) | 1.19 (0.82-1.72) |
| History of diabetes |  |  |  |  |
| No | 318 | 71 (22.3) | Ref. | Ref. |
| Yes | 95 | 17 (17.9) | 0.80 (0.50-1.29) | 0.81 (0.50-1.30) |
| History of hypertension |  |  |  |  |
| No | 155 | 31 (20.0) | Ref. | Ref. |
| Yes | 259 | 57 (22.0) | 1.10 (0.75-1.63) | 1.08 (0.72-1.62) |
| History of cardiovascular disease |  |  |  |  |
| No | 325 | 70 (21.5) | Ref. | Ref. |
| Yes | 86 | 17 (19.8) | 0.92 (0.57-1.47) | 0.90 (0.57-1.43) |
| History of liver disease |  |  |  |  |
| No | 353 | 73 (20.7) | Ref. | Ref. |
| Yes | 60 | 14 (23.3) | 1.13 (0.68-1.87) | 1.20 (0.73-1.97) |
| History of cancer |  |  |  |  |
| No | 380 | 85 (22.4) | Ref. | Ref. |
| Yes | 31 | 2 (6.5) | 0.29 (0.07-1.12) | 0.29 (0.08-1.11) |
| History of pulmonary disease |  |  |  |  |
| No | 299 | 66 (22.1) | Ref | Ref. |
| Yes | 114 | 22 (19.3) | 0.87 (0.57-1.35) | 1.00 (0.65-1.55) |
| Received ≥1 COVID-19 dose |  |  |  |  |
| No | 211 | 41 (19.4) | Ref. | Ref. |
| Yes | 193 | 44 (22.8) | 1.17 (0.80-1.71) | 1.27 (0.82-1.96) |

* Adjusted prevalence ratios were estimated by modified Poisson regression with robust variance estimators; a separate model was used for each covariate shown and all models included adjustment for calendar period, age, sex, and race and ethnicity.

Abbreviations: HIV, human immunodeficiency virus; MOUD, medications for opioid use disorder; SSP, syringe service programs

Supplemental Table 3. Sensitivity analysis – prevalence and correlates of anti-*spike-1* SARS-CoV-2 IgG antibodies in the general vaccine access era (n = 381).

|  | Total | Prevalence,  n (%) | Crude  Prevalence Ratio  (95% CI) | Adjusted  Prevalence Ratio*  (95% CI) |
| --- | --- | --- | --- | --- |
| Calendar period |  |  |  |  |
| Apr.27.2021-May.2021 | 40 | 26 (65.0) | Ref. | Ref. |
| June.2021-Nov.2021 | 195 | 153 (78.5) | 1.21 (0.95-1.53) | 1.23 (0.98-1.54) |
| Dec.2021-July.2022 | 146 | 125 (85.6) | 1.32 (1.04-1.67) | 1.38 (1.10-1.73) |
| Age group, y |  |  |  |  |
| 18-44 | 40 | 25 (62.5) | 0.70 (0.54-0.89) | 0.75 (0.56-1.01) |
| 45-54 | 80 | 56 (70.0) | 0.78 (0.66-0.92) | 0.81 (0.69-0.95) |
| 55-64 | 173 | 144 (83.2) | 0.93 (0.84-1.02) | 0.93 (0.84-1.03) |
| ≥65 | 88 | 79 (89.8) | Ref. | Ref. |
| Sex |  |  |  |  |
| Male | 247 | 202 (81.8) | Ref. | Ref. |
| Female | 134 | 102 (76.1) | 0.93 (0.83-1.04) | 0.95 (0.85-1.06) |
| Race and ethnicity |  |  |  |  |
| Non-Hispanic Black | 315 | 260 (82.5) | Ref. | Ref. |
| Non-Hispanic white | 48 | 30 (62.5) | 0.76 (0.60-0.95) | 0.85 (0.66-1.10) |
| Other including Hispanic | 18 | 14 (77.8) | 0.94 (0.73-1.21) | 1.02 (0.80-1.30) |
| Educational attainment |  |  |  |  |
| Less than H.S. | 211 | 174 (82.5) | Ref. | Ref. |
| Completed H.S./GED or more | 169 | 130 (76.9) | 0.93 (0.84-1.03) | 0.94 (0.85-1.03) |
| Marital status |  |  |  |  |
| Never married | 191 | 149 (78.0) | 0.98 (0.86-1.11) | 1.03 (0.91-1.16) |
| Widowed/divorced | 92 | 77 (83.7) | 1.05 (0.92-1.20) | 1.05 (0.92-1.20) |
| Married | 98 | 78 (79.6) | Ref. | Ref. |
| Lives alone |  |  |  |  |
| No | 221 | 169 (76.5) | Ref. | Ref. |
| Yes | 158 | 133 (84.2) | 1.10 (1.00-1.22) | 1.08 (0.98-1.19) |
| Experienced homelessness (6 mo.) |  |  |  |  |
| No | 332 | 276 (83.1) | Ref. | Ref. |
| Yes | 49 | 28 (57.1) | 0.69 (0.54-0.88) | 0.73 (0.57-0.93) |
| Currently employed |  |  |  |  |
| No | 333 | 264 (79.3) | Ref. | Ref. |
| Yes | 48 | 40 (83.3) | 1.05 (0.92-1.21) | 1.06 (0.92-1.22) |
| Personal income (6 mo.) |  |  |  |  |
| ≥$5,000 | 99 | 85 (85.9) | Ref. | Ref. |
| <$5,000 | 272 | 211 (77.6) | 0.90 (0.82-1.00) | 0.94 (0.85-1.03) |
| Incarcerated (6 mo.) |  |  |  |  |
| No | 373 | 300 (80.4) | Ref. | Ref. |
| Yes | 8 | 4 (50.0) | 0.62 (0.31-1.25) | 0.68 (0.35-1.30) |
| Transactional sex (6 mo.) |  |  |  |  |
| No | 368 | 296 (80.4) | Ref. | Ref. |
| Yes | 13 | 8 (61.5) | 0.77 (0.50-1.18) | 0.81 (0.51-1.27) |
| Hazardous alcohol use (AUDIT-C) |  |  |  |  |
| No | 313 | 251 (80.2) | Ref. | Ref. |
| Yes | 68 | 53 (77.9) | 0.97 (0.85-1.12) | 0.93 (0.81-1.07) |
| Cocaine use (6 mo.) |  |  |  |  |
| No | 261 | 217 (83.1) | Ref. | Ref. |
| Yes | 120 | 87 (72.5) | 0.87 (0.77-0.99) | 0.93 (0.82-1.06) |
| Heroin or fentanyl use (6 mo.) |  |  |  |  |
| No | 248 | 210 (84.7) | Ref. | Ref. |
| Yes | 133 | 94 (70.7) | 0.83 (0.74-0.94) | 0.89 (0.78-1.01) |
| Injection drug use (6 mo.) |  |  |  |  |
| No | 309 | 256 (82.8) | Ref. | Ref. |
| Yes | 72 | 48 (66.7) | 0.80 (0.68-0.95) | 0.88 (0.74-1.06) |
| Attended SSP (6 mo.) |  |  |  |  |
| No | 341 | 277 (81.2) | Ref. | Ref. |
| Yes | 40 | 27 (67.5) | 0.83 (0.67-1.04) | 0.89 (0.70-1.13) |
| Prescribed MOUD (6 mo.) |  |  |  |  |
| No | 206 | 167 (81.1) | Ref. | Ref. |
| Yes | 175 | 137 (78.3) | 0.97 (0.87-1.07) | 1.01 (0.91-1.12) |
| HIV status |  |  |  |  |
| Living without HIV | 257 | 198 (77.0) | Ref. | Ref. |
| Living with HIV | 124 | 106 (85.5) | 1.11 (1.01-1.22) | 1.11 (1.01-1.22) |
| History of diabetes |  |  |  |  |
| No | 295 | 232 (78.6) | Ref. | Ref. |
| Yes | 86 | 72 (83.7) | 1.06 (0.95-1.19) | 1.07 (0.96-1.19) |
| History of hypertension |  |  |  |  |
| No | 152 | 114 (75.0) | Ref. | Ref. |
| Yes | 229 | 190 (83.0) | 1.11 (0.99-1.23) | 1.04 (0.94-1.16) |
| History of cardiovascular disease |  |  |  |  |
| No | 304 | 242 (79.6) | Ref. | Ref. |
| Yes | 76 | 61 (80.3) | 1.01 (0.89-1.14) | 0.98 (0.87-1.12) |
| History of liver disease |  |  |  |  |
| No | 320 | 258 (80.6) | Ref. | Ref. |
| Yes | 58 | 44 (75.9) | 0.94 (0.81-1.10) | 0.92 (0.79-1.07) |
| History of cancer |  |  |  |  |
| No | 349 | 274 (78.5) | Ref. | Ref. |
| Yes | 32 | 30 (93.8) | 1.19 (1.07-1.33) | 1.15 (1.04-1.26) |
| History of pulmonary disease |  |  |  |  |
| No | 279 | 219 (78.5) | Ref | Ref. |
| Yes | 100 | 85 (85.0) | 1.08 (0.98-1.20) | 1.06 (0.96-1.17) |
| Received ≥1 COVID-19 vaccine dose |  |  |  |  |
| No | 92 | 38 (41.3) | Ref. | Ref. |
| Yes | 272 | 253 (93.0) | 2.25 (1.76-2.88) | 2.16 (1.69-2.77) |

* Adjusted prevalence ratios were estimated by modified Poisson regression with robust variance estimators; a separate model was used for each covariate shown and all models included adjustment for calendar period, age, sex, and race and ethnicity.

Abbreviations: HIV, human immunodeficiency virus; MOUD, medications for opioid use disorder; SSP, syringe service programs
